# Soil Organic Carbon Pools and Stocks in Permafrost-Affected Soils on the Tibetan Plateau

**DOI:** 10.1371/journal.pone.0057024

**Published:** 2013-02-26

**Authors:** Corina Dörfer, Peter Kühn, Frank Baumann, Jin-Sheng He, Thomas Scholten

**Affiliations:** 1 Department of Geosciences, Physical Geography and Soil Science, University of Tuebingen, Tuebingen, Germany; 2 Department of Ecology, College of Urban and Environmental Sciences, Peking University, Beijing, People’s Republic of China; Utrecht University, Netherlands

## Abstract

The Tibetan Plateau reacts particularly sensitively to possible effects of climate change. Approximately two thirds of the total area is affected by permafrost. To get a better understanding of the role of permafrost on soil organic carbon pools and stocks, investigations were carried out including both discontinuous (site Huashixia, HUA) and continuous permafrost (site Wudaoliang, WUD). Three organic carbon fractions were isolated using density separation combined with ultrasonic dispersion: the light fractions (<1.6 g cm^−3^) of free particulate organic matter (FPOM) and occluded particulate organic matter (OPOM), plus a heavy fraction (>1.6 g cm^−3^) of mineral associated organic matter (MOM). The fractions were analyzed for C, N, and their portion of organic C. FPOM contained an average SOC content of 252 g kg^−1^. Higher SOC contents (320 g kg^−1^) were found in OPOM while MOM had the lowest SOC contents (29 g kg^−1^). Due to their lower density the easily decomposable fractions FPOM and OPOM contribute 27% (HUA) and 22% (WUD) to the total SOC stocks. In HUA mean SOC stocks (0–30 cm depth) account for 10.4 kg m^−2^, compared to 3.4 kg m^−2^ in WUD. 53% of the SOC is stored in the upper 10 cm in WUD, in HUA only 39%. Highest POM values of 36% occurred in profiles with high soil moisture content. SOC stocks, soil moisture and active layer thickness correlated strongly in discontinuous permafrost while no correlation between SOC stocks and active layer thickness and only a weak relation between soil moisture and SOC stocks could be found in continuous permafrost. Consequently, permafrost-affected soils in discontinuous permafrost environments are susceptible to soil moisture changes due to alterations in quantity and seasonal distribution of precipitation, increasing temperature and therefore evaporation.

## Introduction

The relationship between soil organic carbon (SOC) stocks and site characteristics has been well investigated in the temperate zones at local and regional scale (e.g. [1]–[4]), but much less studies exist about the role of SOC in high-cold alpine regions (e.g. [5]–[8]). Studies in Arctic regions have shown that permafrost-influenced alpine ecosystems are highly sensitive to global climate change [9]. Prevailing low temperatures and permanently low turn-over rates result in large SOC stocks, providing a great emission potential for greenhouse gases such as CO_2_ and CH_4_ [10]–[14]. Thus, an estimate of SOC stocks in their extent and distribution is essential to predict feedback of SOC on global climate change [4], [1], [3]. Furthermore, differentiation between carbon pools is necessary because various SOM fractions show large differences in their turn-over rates according to their mineral binding [15]–[17].

The Tibetan Plateau is a particularly sensitive area in terms of possible effects of global climate change. It is the largest and highest plateau on earth and covers an area of 2.5 million square kilometers with an average altitude of more than 4000 m a.s.l. comprising about a quarter of China’s mainland [18]. On the Tibetan Plateau seasonally frozen soils are widespread [19]. Approximately two thirds of the total area is affected by permafrost [20]. Many studies focus on recent changes of the permafrost conditions on the Tibetan Plateau, attesting increasing permafrost temperatures, active layer thickness and rising degradation (e.g. [21]–[26]. Due to relatively high permafrost temperatures just below the freeze-thaw point, the so-called warm permafrost is more sensitive to global warming than the cold permafrost of higher latitudes [27]. The permafrost distribution is closely related to the characteristics of the land surface such as slope, exposure, vegetation distribution and snow cover. In this study permafrost is classified after the Chinese Permafrost Classification which differs from the classification of the International Permafrost Association (IPA). 50–90% of permafrost is required for classifying discontinuous permafrost after IPA Permafrost Classification; 30–70% is required after Chinese Permafrost Classification [28].

Recent soil ecological research mainly focuses on soil temperature as the main driving force for ecosystem processes (e.g. [3], [29]–[31]). Baumann et al. [6] showed that nutrient availability is a limiting factor for plant growth as well, which in turn is controlled by soil moisture. Furthermore studies show soil moisture is the dominant parameter regarding to the spatial variation of SOC contents [6] and soil CO_2_ efflux [32] on the landscape scale in permafrost influenced ecosystems. Zhao et al. [20] found a negative relationship between vegetation cover/biomass and active layer thickness in alpine meadow ecosystems. Permafrost favors the development of alpine meadow ecosystems, protecting in turn permafrost by their dense vegetation cover from degradation [33], [34].

According to Wang et al. [5] about 33.52 Pg SOC are stored in grassland soils of the Tibetan Plateau down to a depth of 70 cm. Alpine meadow and alpine steppe soils have a share of 23.2 Pg, which represents 2.5% of the global soil carbon pool [5]. Alpine meadows make up 38.2% of total grassland soil carbon in Chinese grassland soils [35]. Wang et al. [33] estimated that the degradation of grassland is resulting in a loss of 57% of SOC in heavy fractions (HF) and 84% in light fractions (LF) from alpine meadow soils in Dari County (Qinghai Province). From 1986 to 2000 land cover changes have led to a loss of 1.8 Gg SOC and a mass loss of 65% in the LF in the upper 30 cm [36].

Major objectives of this study are (1) to investigate SOC stocks and their affiliation to pools by density fractionation and (2) to examine interactions of SOC, soil moisture, and active layer thickness in permafrost-affected soils on the Tibetan Plateau. For comparing stocks and processes in soils affected by continuous and discontinuous permafrost adequately, sites with similar rainfall under varying temperatures were selected.

## Materials and Methods

### Study Sites

**Figure 1:**
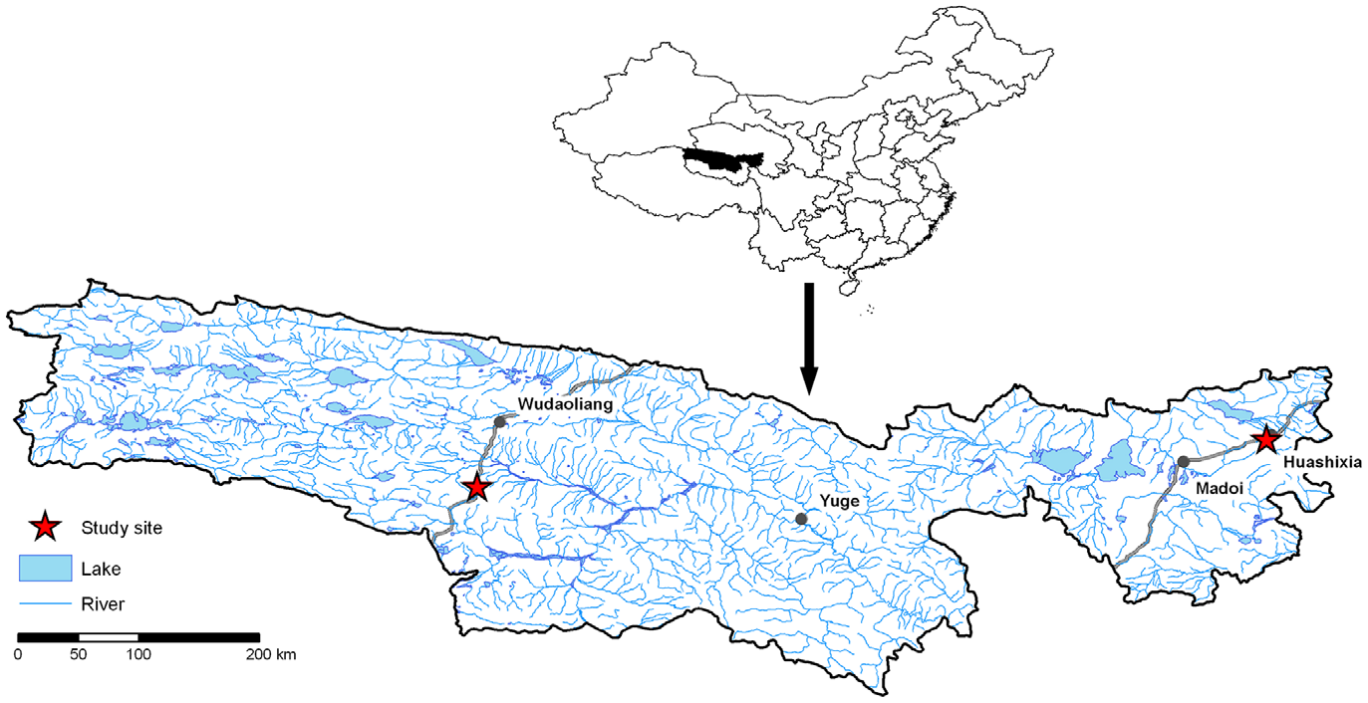
Location of the study sites on the Tibetan Plateau.

The study sites are located on the northeastern Tibetan Plateau, Qinghai Province, China and were investigated in May/June 2009 and 2010 (Fig. 1). Site HUA is situated near the settlement of Huashixia in Maduo County in the Yellow River catchment area, 4300 m a.s.l. The area is affected by the subtropical East Asian Monsoon, which transports air masses with high water vapor content from the lowlands to the Tibetan Plateau through the meridional flow furrows [37], leading to relatively high rainfall. The nearest climate station at Maduo shows a Mean Annual Air Temperature (MAAT) of −4.1°C and a Mean Annual Precipitation (MAP) of 326 mm [38]. The catchment area of the Yellow River (Huang He) is characterized by discontinuous, unstable permafrost. The soils freeze to a depth of 2–3 m, while the upper limit of the permafrost lies in 4–7 m depth; so-called taliks have developed [39]. This vertical disconnection of the permafrost is widespread near the study site. The site is influenced by severe summer grazing with yak *(Bos grunniens)* and sheep *(Ovis aries)* and a temporary settlement by nomads.

Site WUD is located in the headwaters of the Yangtze River, in the middle between Wudaoliang and Tuotuohe next to the Qinghai-Tibet Highway. Precipitation decreases from SE to NW on the Plateau, but due to its location on the Fenghuo Shan mountains, site WUD is characterized by higher precipitation and lower temperatures as the surrounding area (MAP: 348 mm, MAAT: −5.75°C, 4801 m a.s.l., [32]). The high elevation and the low influence of the South Asian Monsoon are responsible for the widespread continuous permafrost in that area [40] which is relatively poor in ice [39]. This also results in a shorter vegetation growth period compared to HUA. During the field campaign no grazing occurred. Nevertheless, the area is used only as late summer pasture due to extreme climate conditions. Generally, grazing is less intensive compared to HUA.

The soils in both study areas are developed in sandy loess, mixed with coarser material derived from frost weathering processes. Poorly developed soils at site HUA are classified as gleyic Fluvisols, haplic Regosols and mollic Cryosols, whereas cambic Cryosols are common in WUD [41]. Alpine *Kobresia* meadow ecosystems are the most common vegetation types on the plateau [38] occurring at elevations ranging from 3200 to 5200 m a.s.l. [42]. At the study sites, particularly *Kobresia pygmaea* and *K. humilis* are widespread. Plant composition is similar at both sites, differing along the altitudinal gradient according to water supply. Strongly rooted, partly felty topsoils are common [43].

### Field Methods

During May and June in 2009, 11 soil profiles were sampled (HUA: 6, WUD: 5). All soil profiles were arranged along an elevation gradient and affected by permafrost with an active layer thickness less than 100 cm at both sites. At each site two soil profiles in footslope position, two soil profiles in lower mid-slope position and two (HUA) and one (WUD) soil profiles in upper mid-slope position were sampled. We set a high value on the comparability of both study sites in topographic position of the soil profiles, inclination of the slopes and vegetative cover. Soil sampling was split into three parts: horizon-wise sampling for pedogenesis and soil chemical analyses using soil pits, schematic sampling conducted by drilling at four depth-increments (0–5, 5–10, 10–20 and 20–30 cm, four replicates each) for C analysis and volumetric sampling at the same depths for bulk density and gravimetric water content determination (three replicates each). Detailed description of soil profiles and pedogenic implications will be published elsewhere.

### Laboratory Analysis

All soil samples were air-dried and sieved to <2 mm. The pH of the samples was measured in deionized water and in 0.01 M CaCl_2_ solution at a solution:soil ratio of 2.5∶1 with a glass electrode (Sentix 81, WTW, pH 340). CaCO_3_ was analyzed gasvolumetrically on ground subsamples (Calcimeter, Eijkelkamp). Total C and N in bulk soil samples and density fractions were determined on ground subsamples by heat combustion with a CNS analyzer (Vario EL III, Elementar GmbH, Hanau, Germany). SOC in bulk soils and density fractions was calculated as the difference between soil total and soil inorganic carbon. Water content was determined by gravimetric water content analysis, corrected by the skeleton content (>2 mm).

Density fractionation was carried out using sodium polytungstate, following the procedures of Grünewald et al. [44]. It is generally accepted that the density of OM is <1.5 g cm^−3^. After Golchin et al. [45] a density fractionation at 1.6 g cm^−3^ separates light organic fractions from mineral dominated heavy fractions under the assumption, that most mineral particles contain less than 20% OM [46]. Three fractions were isolated: the light fractions free particulate organic matter (FPOM) and occluded particulate organic matter (OPOM) with a density <1.6 g cm^−3^, plus a heavy fraction of mineral associated organic matter (MOM) with a density of >1.6 g cm^−3^. FPOM was separated by floatation after gently shaking in a sodium polytungstate solution (soil:solution ratio 1∶5) and centrifugation at 4,500 rpm for 20 min, followed by vacuum filtration. OPOM was separated after ultrasonic dispersion (58 J ml^−1^) and centrifugation at 4,500 rpm for 15 min. Calibration of the ultrasonic output energy was carried out according to Roscoe et al. [47]. The remains (MOM) were washed three times to remove the salt. Due to soil inhomogeneity fractionation was carried out twice per sample. The dried and ground fractions were analyzed for C, N and SOC. The density solution was recycled after Six et al. [48]. SOC stocks for bulk soils and individual fractions were calculated down to a depth of 30 cm, according to Ohtsuka et al. [7]:
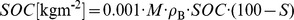
where M is the soil layer thickness, 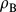 is the bulk density (g cm^−3^) of the soil, SOC is the soil organic carbon content (Mass%) and S is the skeleton content (Mass%).

## Results

### Soil Organic Carbon Content and SOC/N Ratios

**Figure 2:**
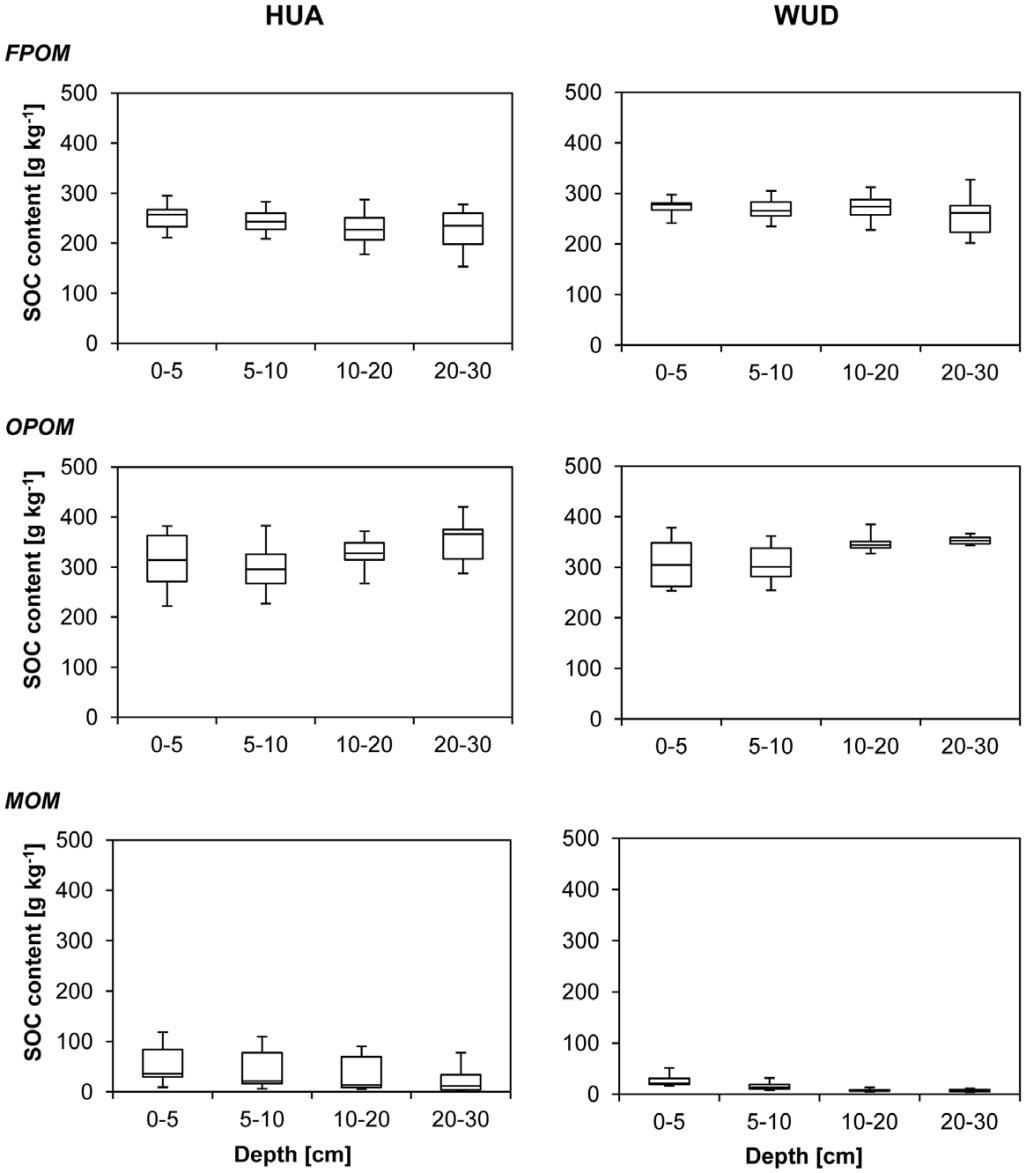
Fractional soil organic carbon (SOC) contents of four different depth increments at Huashixia (HUA, left, n = 40) und Wudaoliang (WUD, right, n = 32).

The SOC content of bulk soil decreased with increasing depth at both sites (Fig. 2). In HUA (51 g kg^−1^) significantly higher mean values were reached than in WUD (19 g kg^−1^). Highest contents occurred in the OPOM fractions (320 g kg^−1^), FPOM followed with 252 g kg^−1^, while they were lowest in the MOM fractions (29 g kg^−1^). The SOC contents in the FPOM decreased with depth, while there were increasing SOC values in the OPOM fractions. The SOC contents of the MOM fraction follow the same depth gradient as SOC in bulk soils at both sites. Significantly higher SOC contents were found in this fraction on site HUA, showing a large variation. The mean recovery of SOC after density fractionation was 95%.

SOC/N ratios of the bulk soils were relatively similar at both sites and decreased with depth (from 12 to 9) – except in extremely moist soils in HUA, where the SOC/N-ratios remained constant (13). The SOC/N ratios of the three fractions were remarkably different. Highest ratios were found in FPOM with a mean ranging from 15–36, followed by OPOM with 14–29. Generally, lower SOC/N ratios occurred in MOM fractions (3–12). We observed slightly rising SOC/N ratios with depth for the POM fractions in all soils, whereas ratios varied less with depth for the MOM fractions.

### Soil Organic Carbon Stocks of Bulk Soil and Fractions

Total SOC stocks in HUA ranged from 1.9 kg m^−2^ to 19.3 kg m^−2^ up to 30 cm depth with a mean of 10.4 kg m^−2^ (Tab. 1). Significantly lower stocks were found in WUD ranging from 2.5 kg m^−2^ to 5.0 kg m^−2^ (mean: 3.4 kg m^−2^).

**Table 1:** Max, Min, Mean values and standard deviations of total (A) and fractional (B) soil organic carbon stocks.

		HUA				WUD			
Depth [cm]		FPOM	OPOM	MOM	Σ OM	FPOM	OPOM	MOM	Σ OM
*A. Total soil organic carbon stocks*									
0–30	Mean [kg m^−2^]SD	1.9(2.1)	0.9(0.9)	7.6(4.5)	10.4(7.1)	0.5(0.2)	0.3(0.1)	2.7(0.6)	3.4(0.8)
0–30	Max [kg m^−2^]	5.0	2.4	12.9	19.3	0.7	0.4	3.9	5.0
0–30	Min [kg m^−2^]	0.2	0.1	1.5	1.9	0.2	0.2	2.2	2.5
*B. Fractional soil organic carbon stocks*									
0–5	Mean [kg m^−2^]SD	0.41(0.25)	0.10(0.05)	1.62(0.58)	2.15(0.83)	0.18(0.10)	0.08(0.03)	0.87(0.19)	1.14(0.29)
5–10	Mean [kg m^−2^]SD	0.39(0.38)	0.16(0.13)	1.34(0.79)	1.90(1.24)	0.08(0.04)	0.05(0.01)	0.50(0.16)	0.64(0.20)
10–20	Mean [kg m^−2^]SD	0.58(0.75)	0.26(0.31)	2.45(1.77)	3.30(2.60)	0.12(0.08)	0.08(0.03)	0.65(0.27)	0.87(0.37)
20–30	Mean [kg m^−2^]SD	0.56(0.80)	0.36(0.46)	2.14(1.71)	3.07(2.88)	0.08(0.05)	0.07(0.02)	0.57(0.18)	0.73(0.24)

HUA: Huashixia (A: n = 24; B: n = 7), WUD: Wudaoliang (A: n = 20; B: n = 6). FPOM: free particulate organic matter, OPOM: occluded particulate organic matter, MOM: mineral-associated organic matter, Σ OM: Total organic matter. SD: standard deviation.

In line with the higher SOC contents, soils in HUA (0.41 kg m^−2^) showed twice as high stocks as in WUD in the top 5 cm (Tab. 1). Fractional SOC stocks in particular depths were highest in the MOM fraction. In WUD, stocks decreased with depth for all three fractions whereas increasing SOC stocks in the OPOM fraction in 20–30 cm depth were evident compared to 10–20 cm depth at HUA.

**Figure 3:**
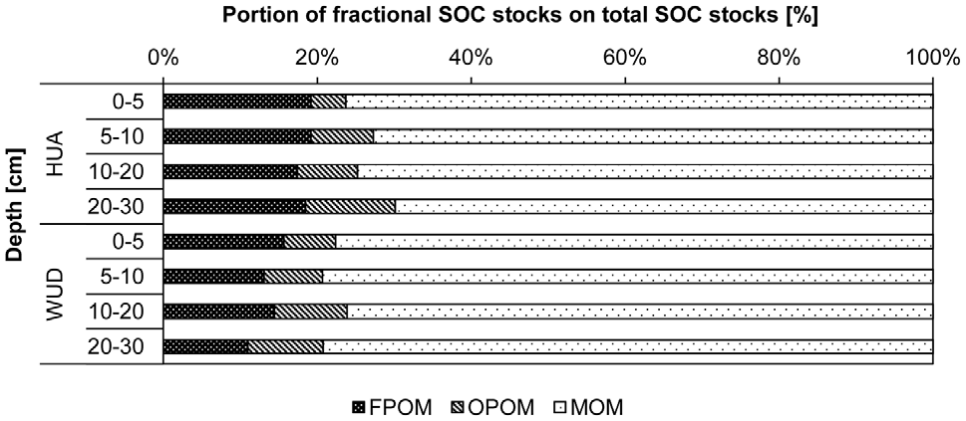
Portion of fractional soil organic carbon (SOC) stocks on total soil organic carbon stocks in particular depth at Huashixia (HUA, top) and Wudaoliang (WUD, bottom).

Variations of the SOC stocks were much higher in HUA than in WUD, especially in the light POM fractions (Tab. 1).The POM fractions in HUA and WUD contributed 27% and 22% to the SOC stocks with 8% in the OPOM fractions at both sites (Fig. 3). Comprising the different depth levels, 53% of SOC is stored in the upper 10 cm in WUD. In HUA only 39% is stored in the upper 10 cm and the portion of FPOM on SOC stocks remained constant with depth, while the portion of OPOM increased. The portion of FPOM on SOC stocks decreased slightly, whereas OPOM stocks increased with depth in WUD. The portion of MOM stock remained relatively constant at 77%, thus slightly higher than in HUA.

### Correlations between Soil Organic Carbon Stocks, Active Layer Thickness and Soil Moisture

**Figure 4:**
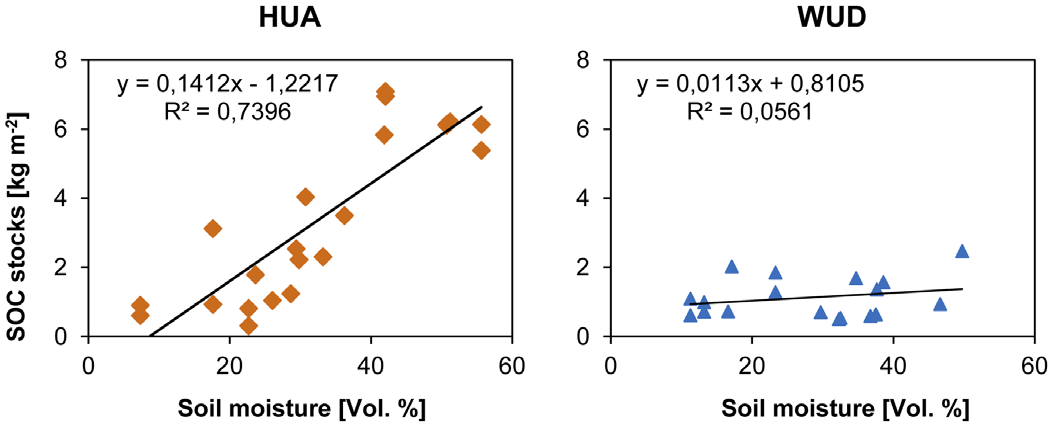
Correlation between soil organic carbon (SOC) stocks and soil moisture in particular depth at Huashixia (HUA, left, n = 24) and Wudaoliang (WUD, right, n = 20).

To assess the influence of soil hydrological properties on SOC stocks, active layer thickness and soil moisture content were taken into account. Since we did not reach the maximum active layer depth in May/June 2009, we used active layer and corresponding soil moisture data from August/September 2011. A significant correlation between SOC stocks and soil moisture can be confirmed for both study sites (Fig. 4). Soil moisture in HUA varies from 7 to almost 56 Vol. % with a high correlation of R^2^ = 0.74 between SOC stocks and soil moisture. The range in WUD is smaller with values between 11 and 50 Vol. % soil moisture and a weak correlation (R^2^ = 0.05).

**Figure 5:**
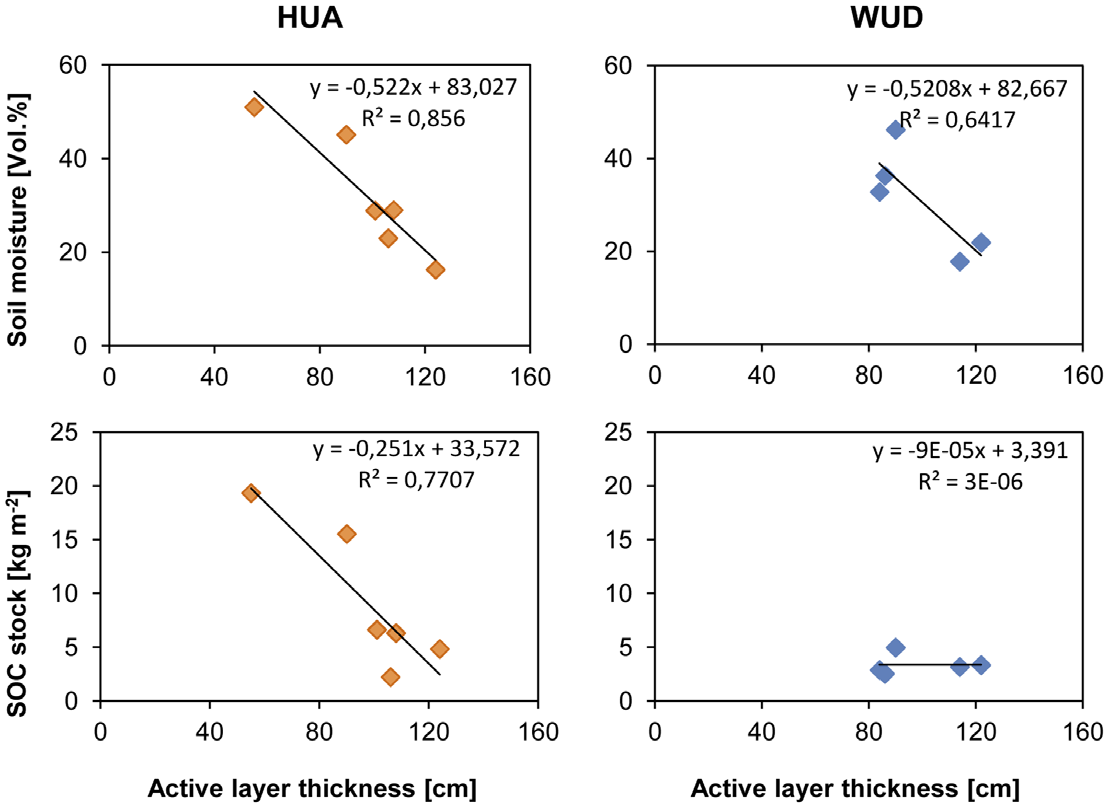
Correlation between soil moisture and active layer thickness (top) and between soil organic carbon (SOC) stock and active layer thickness (bottom) at Huashixia (HUA, left, n = 6) and Wudaoliang (WUD, right, n = 5).

An inverse correlation can be observed for active layer thickness and soil moisture (Fig. 5). The mean thaw depth at both locations is similar (HUA: 97 cm, WUD: 99 cm). Contrarily, the range differs distinctly, with a variation coefficient of 22.0 in HUA and 15.8 in WUD. The interrelation between active layer thickness and SOC stocks is positive for site HUA with R^2^ = 0.77. For site WUD, similar significant correlations could not be detected.

## Discussion

### Soil Organic Carbon Content

SOC contents decreased with soil depth at both locations in all soil profiles. At site HUA, however, much higher overall values were found than in WUD. The more humid, partly water logging conditions inhibit microbial decomposition processes [49] leading in combination with a more dense vegetation cover to a larger accumulation of organic matter.

Concerning the relative amount of OM fractions, our findings are in line with the results of Golchin et al. [50], who found higher SOC contents in the OPOM than in the FPOM and MOM fractions as well. Wang et al. [33] isolated a light fraction (LF) and a heavy fraction (HF) using a 1.8 g cm^−3^ density solution. In LF SOC contents of 290 g kg^−1^ (0–10 cm) and 260 g kg^−1^ (10–20 cm), for HF 30 g kg^−1^ (0–10 cm) and 40 g kg^−1^ (10–20 cm) were found. The comparably slightly lower SOC contents in the LF may reflect the influence of the MOM fraction. However, the comparison is difficult, because different density ranges for the fractionation were used [33].

SOC in FPOM and MOM decreased with depth, whereas the contents of the OPOM fractions increased slightly at both sites. Larger aggregates composed of coarse textured OPOM with a lower degree of decomposition were present in the depth increment 0–10 cm. A reduction of particle size with depth was clearly observable during the fractionation process. Even though the SOC content was relatively small compared with the POM fractions, the portion of total SOC stored in OPOM is large (Fig. 2).

### OC/N Ratios

OC/N ratios of bulk soil decreased with depth indicating a higher age and grade of humification in the subsoil [51]. SOC/N ratios in water-saturated soils in HUA remained relatively stable with depth indicating inhibited decomposition processes. The highest SOC/N ratios with depth were found in the FPOM fraction followed by OPOM and MOM. Comparable results were reported by Grünewald et al. [44] and John et al. [52], who also observed decreasing SOC/N ratios with depth from FPOM>OPOM>MOM for all soils, indicating an increasing degree of OM degradation and humification. Golchin et al. [50] observed higher ratios for OPOM than for FPOM, whereas Kölbl and Kögel-Knabner [53] found no differences between both fractions.

OC/N ratios in FPOM showed little variations with depth, while the portion of carbon content decreases. Further, we observed a decrease in particle size in the POM fractions during the fractionation that may also contribute to a shift in the SOC/N ratio with depth. The very low SOC/N ratios in MOM fractions suggest a generally larger contribution of microbial biomass and, hence, stronger microbial decomposition of plant debris than for POM fractions. The contribution of inorganic N to the total N content – resulting in a very low SOC/N ratio – cannot be excluded.

### Soil and Fractional Organic Carbon Stocks

Our results on SOC stocks are in line with other published data on alpine meadow soils on the Tibetan Plateau [54], [19], [7], [55] as well as on tundra soils in Siberia [56] at a depth of 0–30 cm (Tab. 2). In HUA mean SOC stocks of 10.4 kg m^−2^ were found, in WUD comparably low 3.4 kg m^−2^ (0–30 cm). Due to small-scale differences in substrate, soil moisture and hence vegetation cover, carbon stocks differ more in HUA than in WUD. Highest stocks occurred in water saturated profiles at HUA (19.3 kg m^−3^) whereas lowest stocks were found in dry profiles with a lower vegetation density in WUD (2.7 kg m^−3^). Our results show that high soil moistures combined with low soil temperatures (due to the isolating effect of dense vegetation) lead to an increased accumulation of soil organic matter [57] and therefore higher SOC stocks than in drier soil profiles. SOC stocks decreased with depth, especially in WUD (Tab. 1).

**Table 2:** Comparison of soil organic carbon (SOC) stocks in high-altitude and high-latitude permafrost-affected ecosystems.

Study	Mean SOC stocks [kg m^−2^]	Depth [cm]	Ecosystem type	Region
Post et al. (1982) [4]	21.8	100	Tundra	
Gundelwein et al. (2007) [59]	30.7	100	Tussock Tundra	Siberia, Russia
Jobbágy and Jackson (2000) [1]	14.2	100	Tundra	Canada
Uhlirova et al. (2007) [56]	16.3	30	Tussock Tundra	Siberia, Russia
Wang et al. (2008) [54]	9.3	30	Alpine steppe	Tibetan Plateau, China
	9.8	30	Alpine meadow	Tibetan Plateau, China
	10.7	30	Alpine swamp meadow	Tibetan Plateau, China
Yang et al. (2008) [19]	6.2	30	Alpine meadow	Tibetan Plateau, China
Ohtsuka et al. (2008) [7]	2.6 to 13.7	30	Alpine meadow	Tibetan Plateau, China
Wang et al. (2002) [5]	53.1	75	Alpine meadow	Qinghai, China
	29.0	75	Alpine meadow	Tibet, China
Yang et al. (2010) [26]	9.2	40	Alpine meadow	Tibetan Plateau, China
	12.4	100	Alpine meadow	Tibetan Plateau, China
This study	3.4 to 10.4	30	Alpine meadow	Tibetan Plateau, China

The comparison of fractional SOC stocks with the results of other published research is challenging, as different density ranges and fractionation methods are used and the number of studies on the Tibetan Plateau is limited. Compared to other grassland ecosystems like steppe soils in Ukraine and Kazakhstan [58] and grassland soils in Saxony-Anhalt, Germany [52], in this study significantly higher portions of FPOM and OPOM on SOC stocks were found. About 18% of the total contents in HUA and 14% in WUD were contributed by the FPOM fraction. The OPOM portions were the same at both sites (8%) and the MOM fractions contributed 74 and 78% to the total SOC stocks (Fig. 3). Due to lower litter production in WUD, lower fraction masses and SOC contents led to lower portions of POM on total SOC stocks (Fig. 3). At site HUA the share of FPOM remained relatively constant with depth, while the stocks in WUD were decreasing. The share of OPOM increased at both sites with increasing depth. The increase in OPOM stocks was linked also to the increasing SOC content of the fractions with increasing depth.

The limitation of water caused a lower turnover of organic matter at very dry profile sites in HUA and WUD. This resulted in a relatively high share of FPOM on SOC with small portions of OPOM similar to desert soils investigated by Kadono et al. [58]. The largest FPOM and OPOM shares were found in water-saturated profiles at site HUA with a contribution of 39% of the POM fractions to the SOC stocks since further degradation of SOM is strongly inhibited there.

Our results are comparable to studies in Siberia [59] and the Tibetan Plateau [36], [33]. Wang et al. [36] isolated a light and heavy fraction (1.85 g cm^−3^ density solution) without distinction between FPOM and OPOM. In this case only trends can be compared with our results. The LF contained 7 kg m^−2^ and thus about 37–44% of SOC from 0–30 cm depth. Altogether, SOC stocks comprised 9.81 kg m^−2^ at a depth of 30 cm. Wang et al. [33] isolated LF and HF with a density range of 1.8 g cm^−3^. They found a SOC stock of 7.5 kg m^−2^ at a depth of 0–20 cm, with 0.8 kg m^−2^ in LF and 2.8 kg m^−2^ in HF in the upper 10 cm. 0.4 kg m^−2^ in LF and 3.5 kg m^−2^ in HF were contained in 10–20 cm depth.

### Correlation between Organic Carbon Stocks, Active Layer Thickness and Soil Moisture

Similar mean active layer thicknesses are evident at both sites, but significantly larger variations were observed in HUA. These spatial dynamics are related to small scale changes in substrate, bulk density, soil moisture values [6] and hence in vegetation coverage leading to larger active layer thicknesses. Patches of dense vegetation have an isolating effect, protecting permafrost from thawing [33], [20] resulting in shallower active layer depths.

The detected thawing depths of 55–124 cm in HUA and 84–122 cm in WUD correspond to the range of the maximum thawing depths of 80–150 cm near WUD in September published by Wang et al. [36]. Yang et al. [19] and Baumann et al. [6] showed, that soil moisture affects significantly extension and distribution of carbon stocks on the Tibetan Plateau, as we found as well. Soil moisture, active layer thickness and carbon stocks correlated strongly at HUA (Fig. 4 and 5). As a consequence of the moist to water-saturated conditions and the dense vegetation cover, a higher amount of organic matter is accumulated in HUA compared to WUD, where a lower litter input due to the shorter growing season and drier conditions accelerating mineralization, are prominent. In WUD we found no correlation between soil moisture and SOC stocks as well as between SOC stocks and active layer thickness.

### Conclusions

In this paper we investigated the interactions of SOC stocks and the proportion of light and heavy SOC fractions with soil moisture and active layer thickness in permafrost-affected soils on the Tibetan Plateau. Furthermore, the affiliation of SOC stocks into different SOC pools was examined. The research sites are located in both continuous (WUD) and discontinuous (HUA) permafrost areas.

SOC stocks, soil moisture, and active layer thickness correlated strongly in discontinuous permafrost, whereas no correlation between SOC stocks and active layer thickness and only a weak relation between SOC stocks and soil moisture could be detected for continuous permafrost. Organic carbon contents and SOC stocks were remarkably lower under continuous permafrost conditions. We conclude that drier soil conditions and a shorter vegetation period compared to areas with discontinuous permafrost account for this. Moreover, these soils contain higher portions of easily decomposable POM fractions.

Although the POM fractions comprise only a small portion of the organic carbon mass balance, they contribute a large proportion on SOC stocks due to their high SOC contents. These results show that different POM fractions play specific roles under the scope of climate change: light POM fractions have short turnover rates and are particularly vulnerable to increasing temperatures in terms of potential CO_2_ and CH_4_ emission from soils.
